# Efficacy and safety of linaclotide plus polyethylene glycol for bowel preparation before colonoscopy in elderly Chinese patients: a systematic review and meta-analysis

**DOI:** 10.3389/fmed.2026.1833539

**Published:** 2026-07-07

**Authors:** Shuaijun Li, Haixing Wei, Jiawei Wang, Xinyue Su, Qing Wu, Jie Wang, Peng Peng, Mengbin Qin

**Affiliations:** Department of Gastroenterology, The Second Affiliated Hospital of Guangxi Medical University, Nanning, China

**Keywords:** bowel preparation, China, colonoscopy, elderly adults, linaclotide, meta-analysis, polyethylene glycol

## Abstract

**Background:**

Elderly adults often have inadequate bowel preparation and poor tolerance to high-volume polyethylene glycol (PEG). Linaclotide may enhance intestinal secretion and transit. We compared linaclotide plus PEG (Lin + PEG) with PEG alone in elderly adults.

**Methods:**

We searched CBM, CNKI, VIP, Chinese Medical Journals Full-text Database, Wanfang, PubMed, Embase, and Web of Science from inception to April 23, 2026, for randomized controlled trials comparing Lin + PEG with PEG alone in Chinese adults aged ≥60 years, or trials reporting extractable elderly subgroup data. Two authors independently screened studies and extracted data. Risk of bias was assessed using Version 2 of the Cochrane risk-of-bias tool for randomized trials (RoB 2). Meta-analysis was performed using Review Manager and Stata. Prespecified subgroup analyses were conducted according to constipation status, linaclotide dose, and PEG-volume comparability.

**Results:**

Twelve RCTs conducted in China, involving 1,334 participants, were included. Lin + PEG improved total bowel cleansing quality [MD = 0.93, 95% CI (0.66–1.20), *p* < 0.00001; I^2^ = 86%] and segmental BBPS scores. Lin + PEG was also associated with lower risks of vomiting [RR = 0.60, 95% CI (0.41–0.86), *p* = 0.006; I^2^ = 0%], abdominal pain [RR = 0.57, 95% CI (0.34–0.94), *p* = 0.03; I^2^ = 10%], abdominal distension [RR = 0.61, 95% CI (0.38–0.98), *p* = 0.04; I^2^ = 50%], and nausea [RR = 0.59, 95% CI (0.38–0.92), *p* = 0.02; I^2^ = 32%]. No significant difference was observed for total adverse events [RR = 0.76, 95% CI (0.57–1.01), *p* = 0.06; I^2^ = 60%]. Lin + PEG also reduced total procedure time [MD = −2.35 min, 95% CI (−3.11 to −1.60), *p* < 0.00001; I^2^ = 68%]. The polyp detection rate (PDR) was higher in the Lin + PEG group [RR = 1.25, 95% CI (1.01–1.55), *p* = 0.04; I^2^ = 47%].

**Conclusion:**

In elderly Chinese adults, Lin + PEG may improve bowel cleansing and reduce total procedure time. No clear increase in reported adverse events was observed, although the certainty of the safety evidence remains limited.

**Systematic review registration:**

https://www.crd.york.ac.uk/PROSPERO/view/CRD420251183002, identifier (CRD420251183002).

## 1 Introduction

Colonoscopy is an essential tool for screening and preventing colorectal cancer (CRC). It is frequently employed to evaluate colorectal symptoms, diagnose conditions, and assess mucosal changes associated with inflammatory bowel disease (1–8). Multidisciplinary guidelines and consensus statements consistently highlight that high-quality colonoscopy necessitates adequate intestinal preparation to ensure comprehensive mucosal exposure and effective lesion detection (4, 9–16). Inadequate preparation diminishes mucosal visibility, prolongs procedure time, and increases the risk of missed lesions, which is a major contributor to post-colonoscopy CRC (4, 17–24).

Colonoscopy is increasingly performed among elderly patients (individuals aged ≥60 years). Multiple systematic reviews and meta-analyses have found that these individuals face a heightened risk of inadequate intestinal preparation (IBP) (25–28). Constipation is notably more prevalent among elderly patients and often coexists with various comorbidities and a decline in functional status (29). Previous research has identified diabetes, ASA grade ≥3, and poor functional status as factors associated with an elevated risk of IBP (30). Consequently, the challenges associated with intestinal preparation for colonoscopy in the elderly arise not from a single factor but from a complex interplay of multiple diseases and diminished gastrointestinal function. Given these multifactorial barriers, conventional high-volume laxatives alone may be insufficient, highlighting the potential role of adjunctive agents such as linaclotide in this vulnerable population. In bowel preparation protocols, high-volume PEG regimens (typically 3–4 L) are generally considered safe in clinical practice; however, their large volume may compromise tolerance and compliance (31). Common adverse effects include nausea, abdominal distension, and vomiting, which can compromise the efficacy of laxatives (2, 4, 32). Additionally, “incomplete intake” has been identified as a significant predictor of IBP (26, 28, 33). Consequently, for elderly, it is essential to make individualized trade-offs between the quality of bowel cleansing and safety tolerance in clinical practice.

Linaclotide is a locally acting guanylate cyclase C (GC-C) receptor agonist that stimulates intestinal fluid secretion and accelerates gastrointestinal transit, thereby improving constipation symptoms (34–38). Furthermore, it has been shown to reduce visceral pain hypersensitivity, effectively alleviating abdominal pain (39). Numerous high-quality randomized controlled trials have demonstrated that linaclotide can enhance defecation frequency, improve fecal consistency, and alleviate abdominal symptoms in patients with chronic constipation and constipation-predominant irritable bowel syndrome (IBS-C). The most common adverse reaction associated with its use is diarrhea (40–44). Based on the aforementioned pharmacological characteristics, linaclotide has been explored in recent years as an adjunctive agent with polyethylene glycol (PEG) for bowel preparation. Randomized controlled trials have indicated that, in general or low-risk populations, low-volume PEG combined with linaclotide (for instance, 1 L PEG plus linaclotide) can alleviate discomfort, such as nausea and vomiting, while preserving the quality of intestinal cleanliness and enhancing the willingness to undergo colonoscopy again. Various studies have also investigated the efficacy of different doses of linaclotide under fractionated regimens (45). However, existing evidence exhibits considerable variability concerning PEG volume, linaclotide dosage, timing of administration, and participant characteristics, including factors such as constipation, advanced age, and the presence of comorbidities. Systematic reviews and meta-analyses suggest a relatively consistent overall benefit; nevertheless, there remains considerable protocol heterogeneity and insufficient evidence for specific populations (46–49). Consequently, we conducted this meta-analysis to compare the efficacy and safety of linaclotide combined with PEG versus PEG alone for intestinal preparation before colonoscopy in elderly Chinese adults.

## 2 Methods

### 2.1 Inclusion and exclusion criteria

The study protocol was registered in PROSPERO (CRD420251183002). Eligible studies were required to meet all of the following criteria: (1) study design: randomized controlled trials (RCTs); (2) population: Chinese adults aged ≥60 years, or trials reporting extractable data for a Chinese elderly subgroup; (3) intervention: bowel preparation using linaclotide combined with polyethylene glycol (Lin + PEG); (4) control: PEG alone; (5) outcomes: studies reporting at least one eligible efficacy or safety outcome, including total BBPS score, segmental BBPS score, adverse events, total procedure time, or polyp detection rate; and (6) data availability: sufficient numerical data were available for quantitative synthesis. Studies were excluded if they met any of the following criteria: (1) duplicate publications or overlapping data sets; (2) non-randomized designs, observational studies, case reports, reviews, conference abstracts without usable data, animal studies, or *in vitro* studies; (3) mixed-age studies without extractable data for participants aged ≥60 years; (4) studies conducted in non-Chinese populations; (5) intervention or control regimens inconsistent with the review question; (6) absence of eligible outcomes; or (7) unavailable full text or insufficient numerical data. The review followed PRISMA guidelines (50–52).

### 2.2 Literature retrieval strategy

The CNKI, VIP, Chinese Medical Journals Full-text Database, Wangfang, PubMed, Embase, and Web of Science databases were systematically searched to identify randomized clinical studies on intestinal preparation in the elderly patients in conjunction with linaclotide. The search period extended from the inception of the databases until April 23, 2026. Additionally, for studies not retrieved through the databases, online searches were performed using platforms such as Google Scholar and the Chinese Clinical Trial Registry. Detailed search strategies for each database are presented in Supplementary Table S1.

### 2.3 Literature screening and data extraction

Two authors (Li and Wang) independently searched the literature for relevant studies and cross-verified their findings. Any discrepancies were resolved through discussion or consultation with a third author (Wei). During the literature search, the title of each article was initially reviewed. After discarding obviously irrelevant studies, the abstract and full text were examined to determine the eligibility for inclusion. Data were extracted independently by the two authors using a standardized data extraction form. The extracted data included the following: (1) Basic information such as the research title, first author, and publication year; (2) Baseline characteristics of the subjects and the intervention measures; (3) Key elements for assessing the risk of bias; (4) Outcome indicators and relevant measurement data. For multi-arm trials, only one intervention arm per study was entered into the quantitative synthesis to avoid double-counting of the control group; when multiple intervention arms were available, the arm with a PEG volume identical or closest to that of the control group was selected. Outcome data were extracted according to the definitions reported in the original studies, and only clinically comparable endpoints were pooled.

### 2.4 Risk of bias assessment

Two authors (Li and Wang) independently assessed risk of bias using RoB 2 (50).

### 2.5 Statistical analysis

Meta-analyses were performed using Review Manager (RevMan, version 5.4; The Cochrane Collaboration, London, United Kingdom). Given the small number of included studies (n = 12) and expected clinical/methodological variability (e.g., participant characteristics, intervention protocols, and follow-up), a random-effects model was used as the primary approach to provide conservative pooled estimates. Binary outcomes were pooled as risk ratios (RRs) with 95% confidence intervals (CIs) using the Mantel–Haenszel method, and continuous variables as mean differences (MDs) with 95% CIs using the inverse-variance method (53). Heterogeneity was assessed using the I^2^ statistic and interpreted alongside clinical comparability. Subgroup analyses were prespecified according to clinically relevant factors, including constipation status, linaclotide dose, and PEG-volume comparability. For outcomes, robustness was examined using switching fixed- and random-effects models and leave-one-out sensitivity analyses. Sensitivity analyses and Egger’s regression tests were performed using Stata (version 18.0; StataCorp LLC, College Station, TX, USA). Publication bias was explored by funnel plots and Egger’s regression test when feasible. A two-sided *p* value < 0.05 was considered statistically significant (54–56).

## 3 Results

### 3.1 Baseline characteristics of the included studies

**Figure 1:**
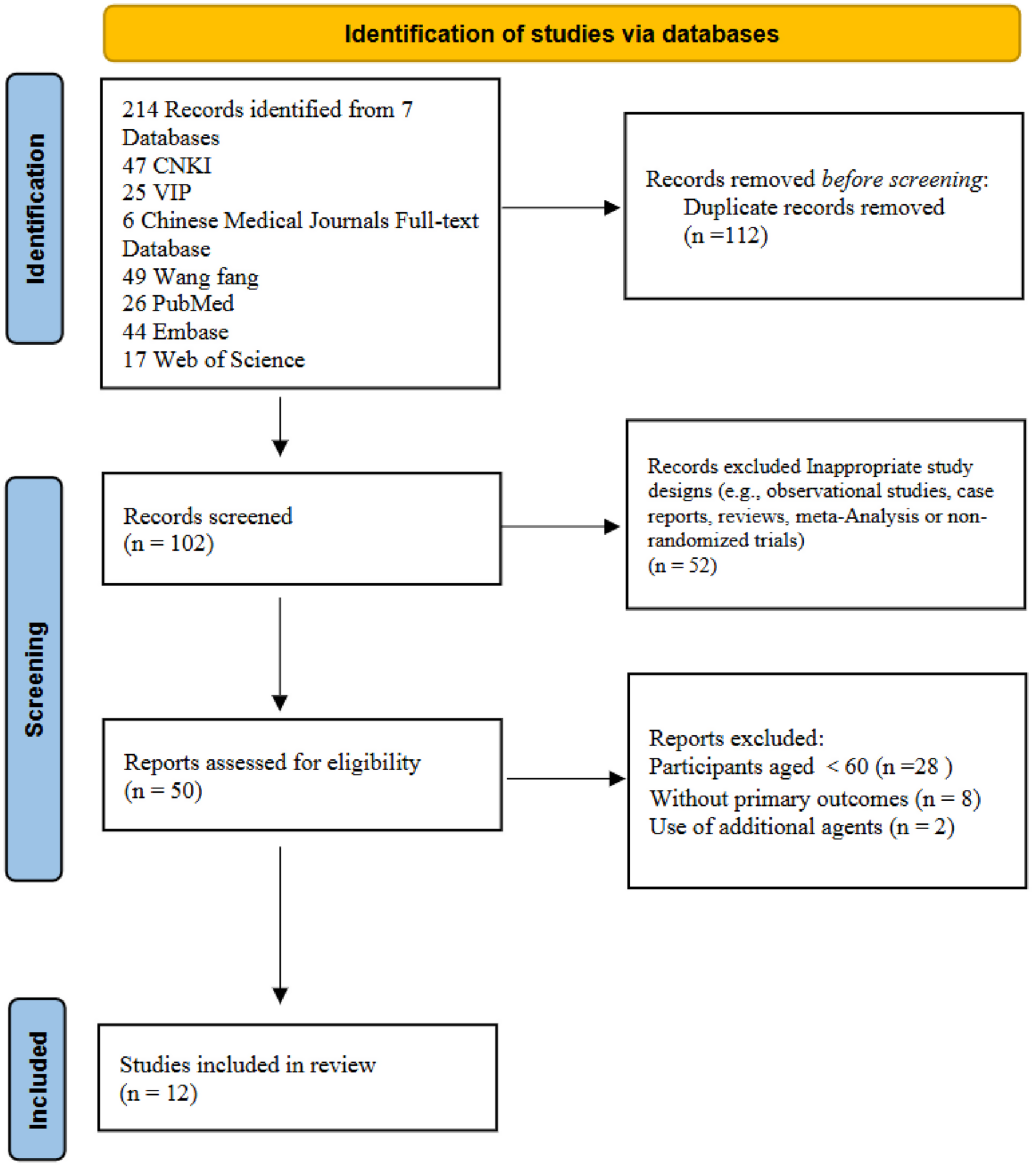
PRISMA flow chart for the systematic search and selection process.

The literature search identified 214 records. After removal of 112 duplicates, 102 records remained for title and abstract screening. Of these, 52 records were excluded because of inappropriate study designs. Fifty full texts were assessed and 38 articles were excluded (Figure 1). Ultimately, twelve studies were included in the meta-analysis (57–68). A total of 1,334 patients participated in these studies, with sample sizes ranging from 50 to 350. Among the 12 RCTs, all were conducted in China. Four were parallel group trials (59, 61, 63, 64), while eight were multi-arm studies (57, 58, 60, 62, 65–68). Ten studies included only elderly participants, whereas the remaining two utilized elderly patients for subgroup analysis (62, 63). Seven studies exclusively focused on patients with constipation, while five did not impose such exclusion criteria (57, 60, 64, 65, 68). The characteristics of the included studies are detailed in Table 1. Intervention details are provided in Supplementary Table S2.

**Table 1:** Baseline characteristics of the included trials.

Author(s)	Year	Study design	Country	Sample size	Methods		Patient condition		Outcome(s)
					Control(s)	Intervention(s)	Age	Constipation	
Zhang et al. (58)	2025	RCT	China	60	3 L PEG	3 L PEG+ 870 μg Lin*	73.4 ± 5.3	All patients	(1)(2)(3)(4)(5)(6)(7)(8)(9)(10)(11)
Xu (63)	2025	RCT	China	85	4 L PEG	3 L PEG+ 870 μg Lin	≥65	All patients	(1)(2)(3)(4)
Tong (60)	2023	RCT	China	108	4 L PEG	2 L PEG+ 580 μg Lin	68.41 ± 4.39	Not required	(1)(2)(3)(4)(6)(7)(8)(9)(10)
Yang (57)	2024	RCT	China	120	3 L PEG	3 L PEG+ 580 μg Lin	68.44 ± 6.23	Not required	(1)(2)(3)(4)(5)
Guo and Fu(61)	2024	RCT	China	84	4 L PEG	4 L PEG+ 870 μg Lin	65.22 ± 8.12	All patients	(1)(2)(3)(4)(5)(6)(7)(8)(9)(10)(11)
Qi et al. (62)	2022	RCT	China	57	3 L PEG	3 L PEG+ 870 μg Lin	68.73 ± 5.72	All patients	(1)(2)(3)(4)
Zhang et al. (59)	2025	RCT	China	90	3 L PEG	3 L PEG+ 870 μg Lin	70.31 ± 2.36	All patients	(1)(2)(3)(4)(5)(6)(7)(8)(11)
Ding (64)	2024	RCT	China	100	3 L PEG	3 L PEG+ 580 μg Lin	66.7 ± 4.7	Not required	(1)(6)(7)(8)(9)(10)(11)
Cheng et al. (67)	2024	RCT	China	100	3 L PEG	2 L PEG+ 870 μg Lin	61.10 ± 5.50	All patients	(1)(7)(8)(9)(10)
Zhang et al. (68)	2023	RCT	China	50	2 L PEG	2 L PEG+ 290 μg Lin	65.76 ± 5.97	Not required	(1)(2)(3)(4)(6)(7)(9)(10)(11)
Sun et al. (65)	2026	RCT	China	350	2 L PEG	2 L PEG+ 290 μg Lin	68.3 ± 6.1	Not required	(1)(6)(7)(9)(10)(11)
Liu et al. (66)	2026	RCT	China	130	3 L PEG	2 L PEG+580 μg Lin	60	All patients	(1)(2)(3)(4)(6)(7)(9)(10)

*Lin = linaclotide (1) Total BBPS score; (2) Left colon BBPS score; (3) Right colon BBPS score; (4) Transverse colon BBPS score; (5) Total procedure time; (6) Polyp detection rate; (7) Vomiting; (8) Abdominal pain; (9) Abdominal distension; (10) Nausea; (11) Total number of adverse events. BBPS – Boston Bowel Preparation Score.

### 3.2 The methodological quality evaluation of the included literature

**Figure 2:**
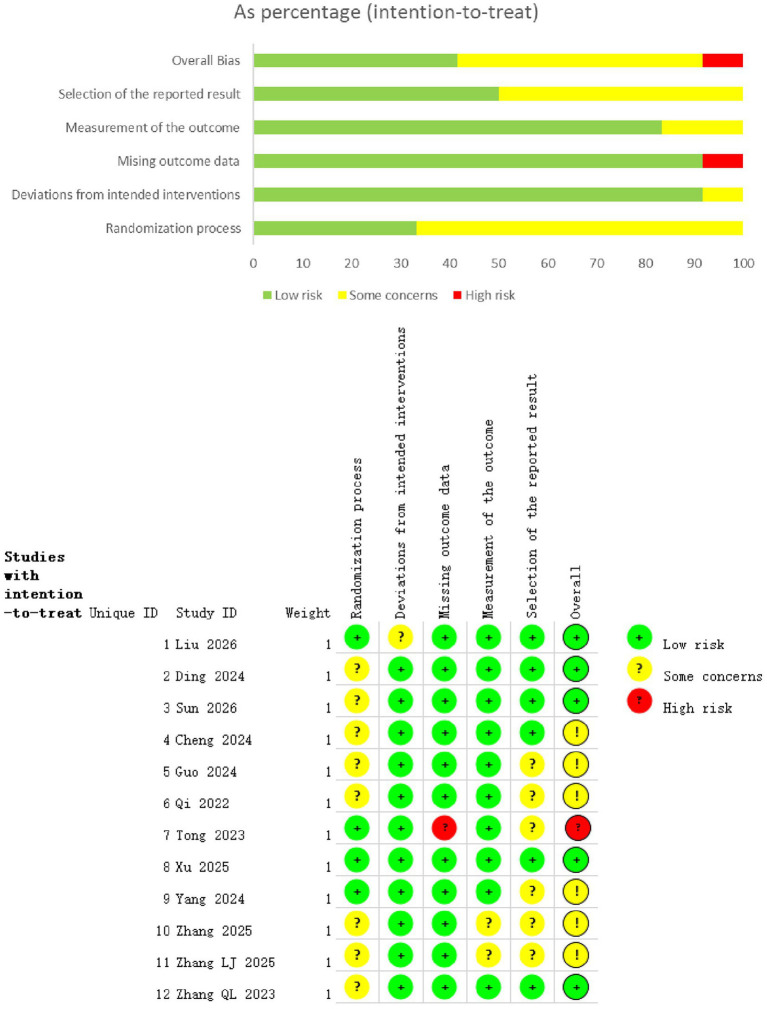
Risk of bias assessment.

Risk of bias was assessed using RoB 2 under the intention-to-treat effect. Twelve randomized controlled trials were included in the assessment. Overall, five studies were judged as low risk of bias, six as having some concerns, and one as high risk. In the domain of the randomization process, several studies were rated as having some concerns because allocation concealment or baseline balance was not sufficiently reported. Most studies were judged as low risk for deviations from intended interventions, measurement of the outcome, and missing outcome data, although two studies were rated as high risk for missing outcome data. In the selection of the reported result domain, half of the studies were judged as having some concerns, mainly because prespecified analysis plans or trial protocols were unavailable. These findings indicate that the overall certainty of the pooled estimates may be affected by incomplete reporting of trial methods and outcome reporting procedures (Figure 2).

### 3.3 Results of meta-analysis

#### 3.3.1 BBPS

**Figure 3:**
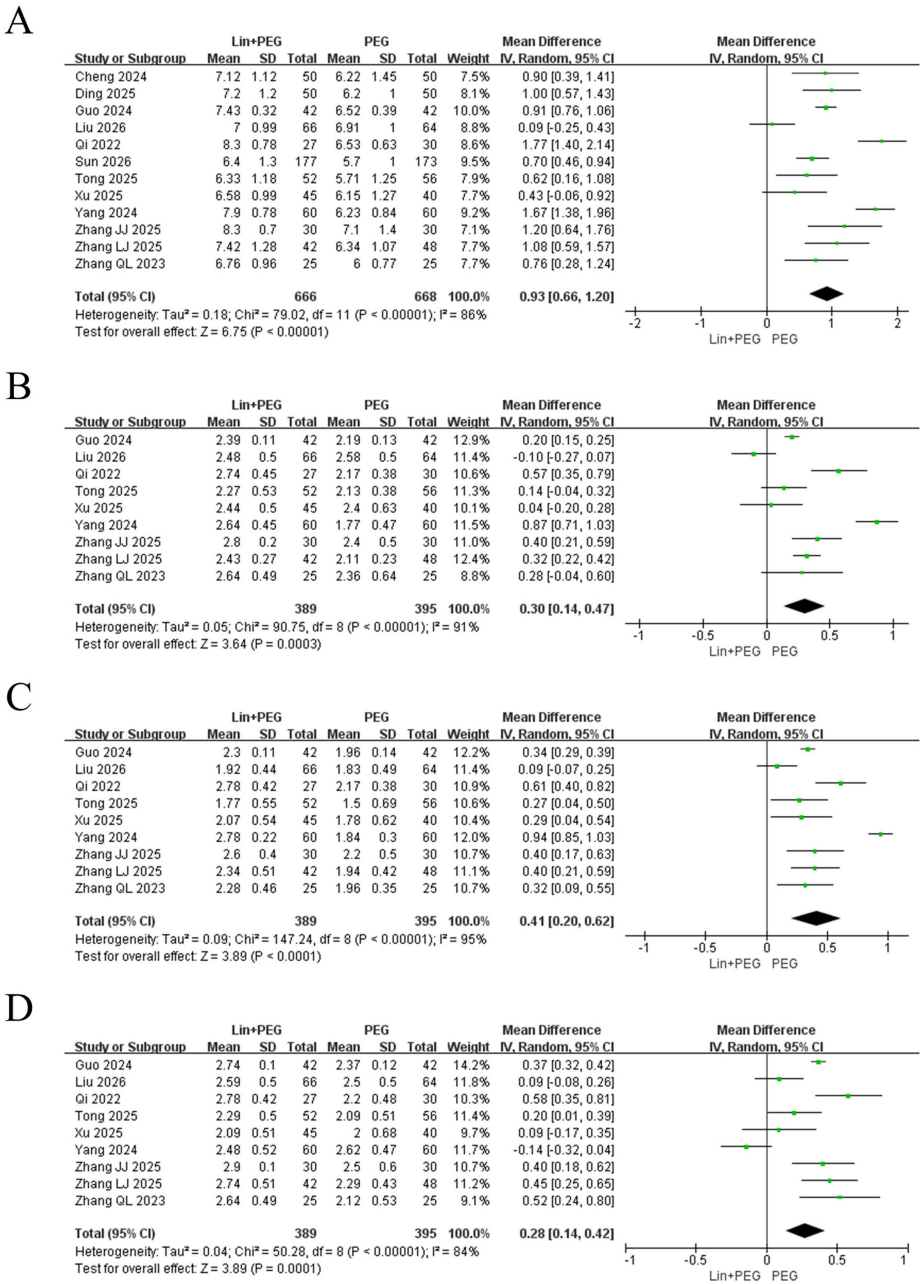
Forest plot comparing the efficacy of bowel preparation. The plot illustrates the mean difference (MD) in bowel preparation scores between the Lin + PEG and PEG groups. Each panel represents a specific component of the Boston Bowel Preparation Scale (BBPS): **(A)** Total BBPS score; **(B)** Left colon BBPS score; **(C)** Right colon BBPS score; and **(D)** Transverse colon BBPS score.

Twelve studies assessed the BBPS. Pooled analyses using a random-effects model showed that Lin + PEG significantly improved bowel cleansing compared with PEG alone, including the total BBPS score [MD = 0.93, 95% CI (0.66–1.20), *p* < 0.00001; I^2^ = 86%]. Nine studies reported segmental BBPS scores, and consistent improvements were observed in the left colon [MD = 0.30, 95% CI (0.14–0.47), *p* = 0.0003; I^2^ = 91%], right colon [MD = 0.41, 95% CI (0.20–0.62), *p* < 0.0001; I^2^ = 95%], and transverse colon [MD = 0.28, 95% CI (0.14–0.42), *p* = 0.0001; I^2^ = 84%] (Figure 3).

#### 3.3.2 Adverse events

**Figure 4:**
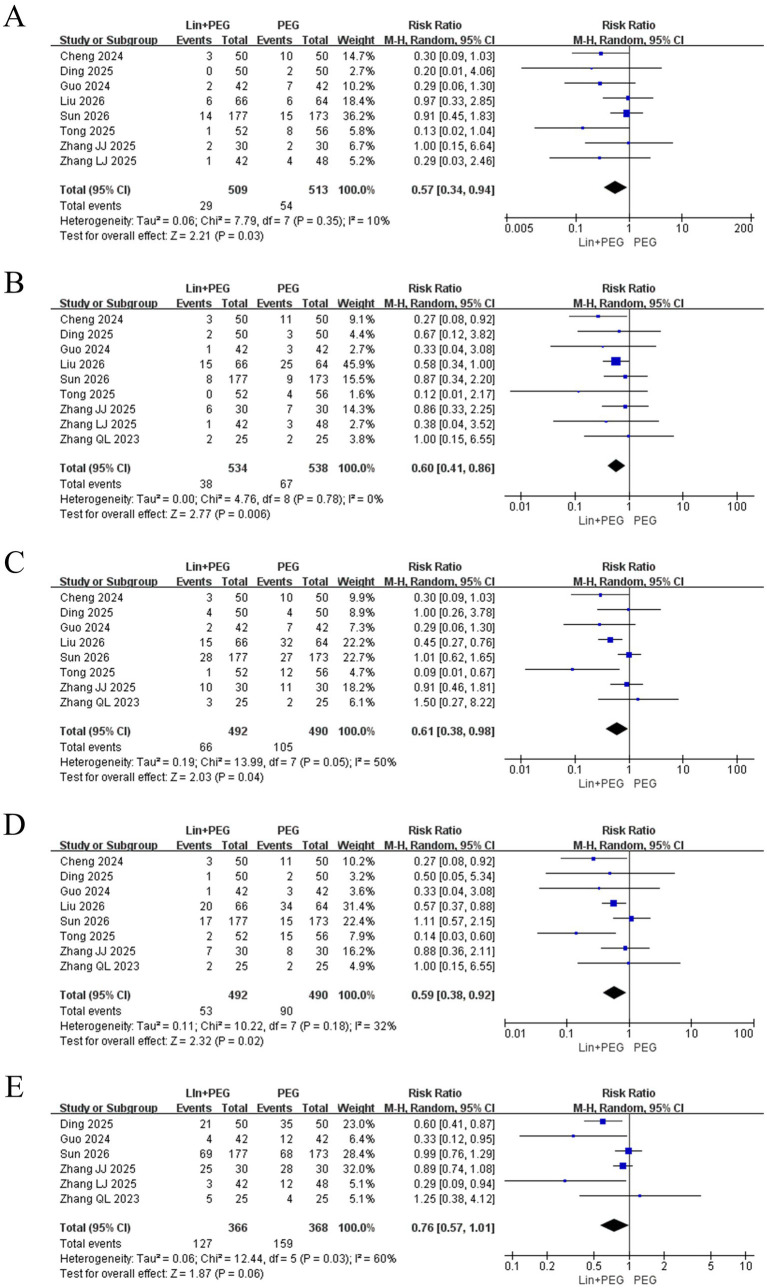
Forest plot of the risk of adverse events for Lin + PEG versus PEG. The plot compares the risk ratio (RR) for various adverse events between the Lin + PEG and PEG groups. Each panel corresponds to a specific adverse event: **(A)** Abdominal pain; **(B)** Vomiting; **(C)** Abdominal distension; **(D)** Nausea; **(E)** Total number of adverse events.

Compared with PEG alone, the Lin + PEG group experienced a significantly lower incidence of individual adverse events, including abdominal pain (RR = 0.57; *p* = 0.03; I^2^ = 10%), vomiting (RR = 0.60; *p* = 0.006; I^2^ = 0%), abdominal distension (RR = 0.61; *p* = 0.04; I^2^ = 50%), and nausea (RR = 0.59; *p* = 0.02; I^2^ = 32%). However, no significant difference was observed between the two groups regarding the total number of adverse events (RR = 0.76; 95% CI [0.57–1.01]; *p* = 0.06; I^2^ = 60%) (Figure 4).

#### 3.3.3 Total procedure time

Three studies reported total procedure time. The pooled analysis showed that Lin + PEG significantly reduced total procedure time compared with PEG alone [MD = −2.35 min, 95% CI (−3.11 to −1.60), *p* < 0.00001; I^2^ = 68%] (Supplementary Figure S1).

#### 3.3.4 PDR

Eight trials reported the PDR, and the pooled analysis demonstrated a statistically significant improvement in the Lin + PEG group compared with PEG alone [RR = 1.25, 95% CI (1.01–1.55), p = 0.04, I^2^ = 47%] (Supplementary Figure S2).

#### 3.3.5 Subgroup analysis

Subgroup analysis according to constipation status showed that Lin + PEG significantly improved the total BBPS score both in elderly patients with constipation [MD = 0.91, 95% CI (0.51–1.30), *p* < 0.00001; I^2^ = 87%] and in studies not restricted to constipation [MD = 0.96, 95% CI (0.52–1.40), *p* < 0.0001; I^2^ = 87%]. No significant subgroup difference was observed [*χ*^2^ = 0.03, df = 1, *p* = 0.86; I^2^ = 0%] (Supplementary Figure S3).

**Figure 5:**
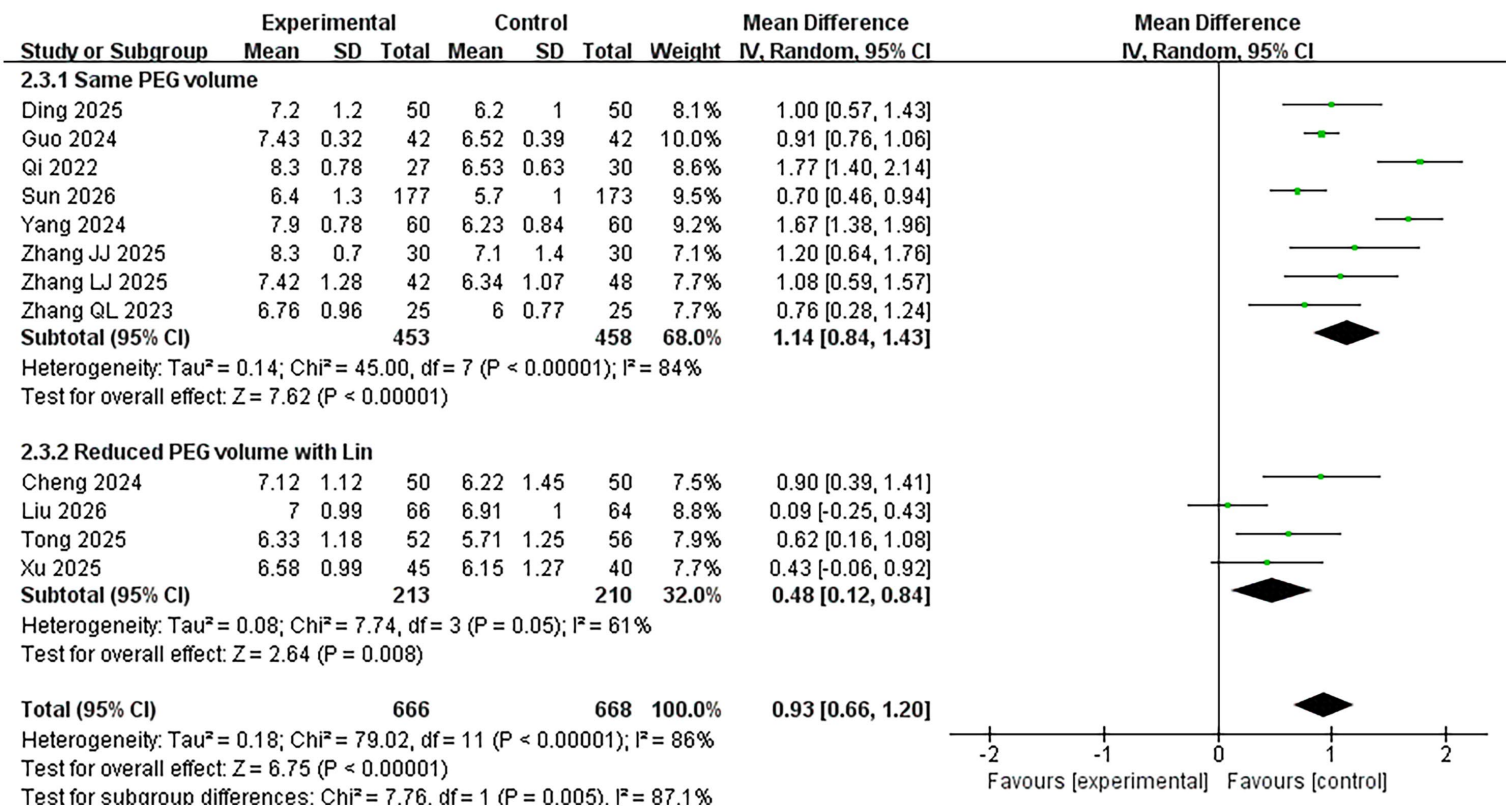
Forest plot of subgroup analysis for total BBPS based on PEG-volume comparability.

Subgroup analysis according to linaclotide dose showed that Lin + PEG significantly improved the total BBPS score in both the ≤580 μg subgroup [MD = 0.81, 95% CI (0.34–1.28), *p* = 0.0007; I^2^ = 91%] and the 870 μg subgroup [MD = 1.06, 95% CI (0.71–1.40), *p* < 0.00001; I^2^ = 79%]. The test for subgroup differences showed no statistically significant difference between the two dose subgroups [*χ*^2^ = 0.68, df = 1, *p* = 0.41; I^2^ = 0%] (Supplementary Figure S4). Subgroup analysis based on PEG-volume comparability showed that Lin + PEG improved the total BBPS score in trials with the same PEG volume between groups [MD = 1.14, 95% CI (0.84–1.43), *p* < 0.00001; I^2^ = 84%] and in trials with reduced PEG volume in the Lin + PEG group [MD = 0.48, 95% CI (0.12–0.84), *p* = 0.008; I^2^ = 61%]. A significant subgroup difference was observed [*χ*^2^ = 7.76, df = 1, *p* = 0.005; I^2^ = 87.1%] (Figure 5), indicating that PEG-volume comparability may influence the estimated effect. The reduced-volume subgroup should be interpreted cautiously because the effect may partly reflect PEG-volume modification rather than linaclotide alone.

#### 3.3.6 Sensitivity analysis

Sensitivity analysis was conducted by switching fixed- and random-effects models, performing leave-one-out analyses, and excluding studies at high risk of bias. The pooled estimates for vomiting, abdominal pain, abdominal distension, nausea, total and segmental cleansing scores (left/right/transverse), total procedure time and PDR remained directionally consistent, indicating robust results. For total adverse events, leave-one-out sensitivity analysis showed that exclusion of Ding (64) changed the pooled estimate to a non-significant result under both fixed-effect and random-effects models, indicating limited robustness of this outcome.

#### 3.3.7 Publication bias

Funnel plots (pseudo 95% confidence limits) and Egger’s regression test, when feasible, were used to determine publication bias. Funnel plots were visually inspected for asymmetry. Egger’s test results were as follows: Total BBPS (*t* = 0.00, *p* = 0.999); left colon BBPS (*t* = −0.69, *p* = 0.512); right colon BBPS (*t* = 0.11, *p* = 0.918); transverse colon BBPS (*t* = 0.85, *p* = 0.422); vomiting (*t* = −0.49, *p* = 0.641); abdominal pain (*t* = −2.10, *p* = 0.080); abdominal distension (*t* = −0.35, *p* = 0.570); nausea (*t* = −0.41, *p* = 0.697); total adverse events (*t* = −1.07, *p* = 0.346); total procedure time (*t* = −0.55, *p* = 0.681); PDR (*t* = 3.33, *p* = 0.016) (Supplementary Figure S5) (see Table 2).

**Table 2:** GRADE summary of findings for Lin + PEG versus PEG alone.

Outcome	Studies (n)	Effect estimate (95% CI)	Certainty	Explicit GRADE downgrades
BBPS, total score	12	MD 0.93 (0.66–1.20)	Moderate	Risk of bias: −1; Inconsistency: −1
BBPS, left colon	9	MD 0.30 (0.14–0.47)	Moderate	Risk of bias: −1; Inconsistency: −1
BBPS, right colon	9	MD 0.41 (0.20–0.62)	Moderate	Risk of bias: −1; Inconsistency: −1
BBPS, transverse colon	9	MD 0.28 (0.14–0.42)	Moderate	Risk of bias: −1; Inconsistency: −1
Vomiting	9	RR 0.60 (0.41–0.86)	Moderate	Risk of bias: −1
Abdominal pain	8	RR 0.57 (0.34–0.94)	Low	Risk of bias: −1; Imprecision: −1
Abdominal distension	8	RR 0.61 (0.38–0.98)	Very low	Risk of bias: −1; Inconsistency: −1; Imprecision: −1
Nausea	8	RR 0.59 (0.38–0.92)	Low	Risk of bias: −1; Imprecision: −1
Total adverse events	6	RR 0.76 (0.57–1.01)	Very low	Risk of bias: −1; Inconsistency: −1; Imprecision: −1
Total procedure time	3	MD − 2.35 min (−3.11 to −1.60)	Low	Risk of bias: −1; Inconsistency: −1
Polyp detection rate	8	RR 1.25 (1.01–1.55)	Very low	Risk of bias: −1; Indirectness: −1; Imprecision: −1

## 4 Discussion

High-quality bowel preparation is essential for the success of colonoscopy, particularly in the elderly patients (9, 69, 70). Due to reduced gastrointestinal motility, high prevalence of chronic constipation, multiple comorbidities, and reduced tolerance to large-volume laxatives, elderly patients experience a significantly higher incidence of inadequate intestinal preparation compared to their younger counterparts (28–30, 71–73). This inadequacy adversely affects mucosal visibility, prolongs procedure duration, and increases the risk of missed diagnoses (74). This meta-analysis systematically assessed the efficacy and safety of linaclotide in conjunction with PEG versus PEG alone for bowel preparation in individuals aged 60 years and older. The available evidence suggests a potential benefit of Lin + PEG for bowel cleansing in elderly patients. Adequate bowel preparation is commonly defined as a total BBPS score ≥6 with all segment scores ≥2; therefore, a mean increase in BBPS may be clinically relevant when baseline preparation quality is close to this threshold, because even modest score improvements may influence whether preparation is judged adequate in practice. However, adequacy rates were not reported consistently or defined uniformly across the included trials and thus could not be synthesized quantitatively. In addition, because BBPS scoring is at least partly observer-dependent, unclear allocation concealment and incomplete blinding may have led to overestimation of the apparent cleansing benefit associated with Lin + PEG. Therefore, although the observed improvement in BBPS is encouraging, its direct impact on downstream clinical decision-making should be interpreted cautiously.

The clinical benefits of adding linaclotide to PEG—improved cleansing and reduced abdominal pain—can likely be explained by its pharmacological profile as a locally acting GC-C receptor agonist. By elevating intracellular cGMP, linaclotide promotes chloride and bicarbonate secretion, which increases luminal fluid, softens feces, and accelerates transit (34, 38, 75). This secretory and prokinetic effect synergistically augments the osmotic load of PEG, enhancing segmental cleansing without requiring larger fluid volumes. Furthermore, we hypothesize that the observed reduction in abdominal pain is driven by two potential mechanisms: (1) accelerated evacuation that relieves luminal distension and mechanical strain, and (2) the release of extracellular cGMP, which may directly dampen colonic nociceptor excitability (39). Elderly patients often struggle to achieve optimal intestinal cleansing solely through osmotic laxatives (76). Linaclotide may synergistically augment the osmotic effects of PEG, thereby improving fecal consistency. While these combined effects theoretically target both retention-driven discomfort and visceral hypersensitivity, this remains a speculative mechanism that warrants further objective investigation.

Safety and tolerability are critical considerations in older adults. Lin + PEG was associated with lower risks of several individual adverse events, including vomiting, abdominal pain, abdominal distension, and nausea. However, these apparent tolerability benefits should be interpreted cautiously because the certainty of evidence was low to very low for several safety outcomes, particularly abdominal distension and total adverse events. In addition, adverse-event definitions and reporting methods were not fully consistent across studies. Therefore, these findings suggest possible, rather than definitive, tolerability advantages of Lin + PEG in elderly patients.

We also analyzed total procedure time and found that the combined protocol significantly reduced this duration (49, 72). This outcome may be attributable to the enhanced overall quality of bowel cleansing achieved with Lin + PEG, which could reduce the need for repeated flushing, aspiration, and other intraprocedural maneuvers (77). However, the absolute reduction was modest (approximately 2 min), which may have limited importance at the individual patient level and should therefore be interpreted as operationally supportive rather than clinically transformative. In addition, procedure time may be influenced not only by bowel cleanliness but also by operator expectations and awareness of treatment allocation (78, 79); thus, unclear allocation concealment and incomplete blinding may also have affected this estimate. Because only a small number of studies reported procedure time and heterogeneity remained present, this finding should be interpreted cautiously.

In this study, Lin + PEG was associated with a higher PDR than PEG alone. Publication-bias assessment suggested potential asymmetry for PDR, which further supports cautious interpretation. However, this finding should be interpreted cautiously because PDR is influenced not only by bowel preparation quality but also by endoscopist performance, withdrawal time, patient risk profile, and lesion prevalence (45). A critical gap is that none of the included trials reported adenoma detection rate (ADR). ADR is the most widely validated colonoscopy quality metric and is directly linked to CRC prevention. In a large community-based cohort, higher ADR was inversely associated with the risks of interval CRC, advanced-stage interval cancer, and fatal interval cancer (24). Therefore, although the observed increase in PDR may suggest a possible benefit, the absence of ADR limits interpretation of the effect of Lin + PEG on meaningful lesion detection. In addition, the certainty of evidence for PDR was very low, which further limits confidence in this finding. Future trials should systematically report lesion-detection outcomes, including both PDR and, preferably, ADR, to clarify whether improved bowel cleansing translates into clinically meaningful detection benefits.

We prespecified subgroup analyses according to clinically relevant factors, including constipation status, linaclotide dose, and PEG-volume comparability. Lin + PEG improved the total BBPS score both in elderly patients with constipation and in studies not restricted to constipation, with no statistically significant subgroup difference. This finding suggests that the observed benefit may not be limited to patients with confirmed constipation, although interpretation remains constrained by differences in study populations and the high heterogeneity within both subgroups. Subgroup analysis by linaclotide dose showed significant improvements in both the ≤580 μg and 870 μg subgroups, without a significant subgroup difference. Therefore, the current evidence does not support a clear dose–response relationship. Because the number of studies in each dose subgroup was limited and within-subgroup heterogeneity remained substantial, the optimal linaclotide dose for bowel preparation in elderly patients cannot be determined from the available evidence.

The subgroup analysis based on PEG-volume comparability provided additional methodological insight. Lin + PEG improved bowel cleansing both in trials with the same PEG volume between groups and in trials with reduced PEG volume in the Lin + PEG group. However, the magnitude of effect was larger in trials using the same PEG volume between groups, and a statistically significant subgroup difference was observed. This finding should be regarded as hypothesis-generating and suggests that PEG-volume comparability may influence the estimated treatment effect. Trials with the same PEG volume are more appropriate for evaluating the adjunctive effect of linaclotide itself, whereas trials with reduced PEG volume in the Lin + PEG group evaluate a combined strategy of linaclotide plus PEG-volume reduction. Therefore, results from reduced-volume comparisons should not be interpreted as evidence of the independent effect of linaclotide alone.

However, several limitations should be acknowledged. First, the number of included studies and the overall sample size were relatively small, and all participants were drawn from the Chinese population, which limits external validity (80). Second, risk-of-bias assessment using RoB 2 showed that several studies had some concerns or high risk of bias. In particular, unclear allocation concealment and incomplete blinding may have influenced effect estimates for observer-dependent outcomes such as BBPS and for procedure-related outcomes such as total procedure time. These limitations may have introduced selection, performance, and detection bias and may have led to overestimation of benefit (81, 82). Third, although PDR was higher in the Lin + PEG group, no included study reported ADR; therefore, the effect of Lin + PEG on clinically meaningful lesion detection remains uncertain. Fourth, linaclotide dose, timing of administration, and PEG regimens were not standardized across studies. In addition, PEG volume differed between groups in some included comparisons, so the pooled effect may partly reflect the combined influence of linaclotide and PEG-volume modification rather than the independent effect of linaclotide alone.

In conclusion, Lin + PEG may improve bowel cleansing quality and selected tolerability outcomes in elderly Chinese patients compared with PEG alone. Although Lin + PEG was associated with a higher PDR, the lack of ADR data limits interpretation of its clinical relevance. The subgroup findings, particularly those related to PEG-volume comparability, should be interpreted as hypothesis-generating rather than definitive because of substantial heterogeneity, small subgroup sizes, and protocol variability. Overall, the findings suggest potential clinical value for Lin + PEG, but the certainty of evidence remains limited by risk of bias, heterogeneous regimens, and restricted generalizability beyond Chinese populations.

## Data Availability

The datasets presented in this study can be found in online repositories. The names of the repository/repositories and accession number(s) can be found in the article/Supplementary material.
