# Supplementary material for: Efficacy and safety of linaclotide plus polyethylene glycol for bowel preparation before colonoscopy in elderly Chinese patients: a systematic review and meta-analysis
Source: Front Med (Lausanne). 2026 Jul 7;13:1833539. doi: 10.3389/fmed.2026.1833539 (PMC13384852; doi:10.3389/fmed.2026.1833539)

| **Database** | **Search strategy** |
| --- | --- |
| Chinese databases(CNKI, VIP, Wanfang, Chinese Medical Journals Full-text Database) | (利那洛肽 OR linaclotide) AND (聚乙二醇 OR 聚乙二醇电解质 OR 复方聚乙二醇电解质 OR PEG OR PEG-ELS) AND (肠道准备 OR 肠道清洁OR 肠道清洗) AND (结肠镜 OR 肠镜检查 OR colonoscopy) Date limit: from database inception to April 23, 2026 |
| PubMed | #1 linaclotide[MeSH Terms] OR linaclotide[Title/Abstract]#2 "Polyethylene Glycol"[MeSH Terms] OR "polyethylene glycol"[Title/Abstract] OR PEG[Title/Abstract] OR "PEG-ELS"[Title/Abstract] OR "polyethylene glycol electrolyte"[Title/Abstract]#3 "Bowel Preparation"[Title/Abstract] OR "bowel cleansing"[Title/Abstract]#4 "Colonoscopy"[MeSH Terms] OR colonoscopy[Title/Abstract] #5 #1 AND #2 AND #3 AND #4 Date limit: up to April 23, 2026 |
| Embase | ('linaclotide'/exp OR linaclotide:ab,ti) AND ('polyethylene glycol'/exp OR 'polyethylene glycol':ab,ti OR peg:ab,ti OR 'peg-els':ab,ti) AND ('bowel preparation':ab,ti OR 'bowel cleansing':ab,ti OR 'intestinal preparation':ab,ti OR 'colon preparation':ab,ti) AND ('colonoscopy'/exp OR colonoscopy:ab,ti) AND ('randomized controlled trial'/exp OR randomized:ab,ti OR rct:ab,ti)Date limit: up to April 23, 2026 |
| Web of Science | TS = (linaclotide AND (polyethylene glycol OR PEG OR "PEG-ELS") AND ("bowel preparation" OR "bowel cleansing") AND (colonoscopy) AND (randomized OR RCT))Date limit: up to April 23, 2026 |

Supplementary Table S1. Detailed search strategies for each database. No language restrictions were applied. Additional manual searches were performed in Google Scholar and the Chinese Clinical Trial Registry (ChiCTR). Reference lists of included studies and relevant reviews were also screened.

| **Study** | **Control PEG regimen** | **Intervention PEG regimen** | **Linaclotide administration timing** |
| --- | --- | --- | --- |
| Zhang JJ et al. | 3 L split-dose: 1 L at 20:00 on the day before colonoscopy, then 2 L 4–6 h before colonoscopy. | Same 3 L PEG split-dose as control. | 290 μg 30 min before breakfast for 3 days before colonoscopy (total 870 μg). |
| Xu et al. | 4 L split-dose: 2 L at 20:00 on the day before colonoscopy, then 2 L 6 h before colonoscopy. | 3 L split-dose: 1 L at 20:00 on the day before colonoscopy, then 2 L 6 h before colonoscopy. | 290 μg 30 min before breakfast for 3 days before colonoscopy (total 870 μg). |
| Tong et al. | 4 L split-dose: 1 L at 20:00 on the day before colonoscopy, then 3 L 6 h before colonoscopy. | 2 L PEG administered 6 h before colonoscopy. | 290 μg 30 min before breakfast on the day before colonoscopy and again 30 min before breakfast on the examination day (total 580 μg). |
| Yang et al. | 3 L split-dose: 1 L at 20:00 on the day before colonoscopy, then 2 L 6 h before colonoscopy. | 3 L split-dose with co-administration of linaclotide: 1 L PEG + 290 μg Lin at 20:00 on the day before colonoscopy, then 2 L PEG + 290 μg Lin 6 h before colonoscopy. | 290 μg at 20:00 on the day before colonoscopy and 290 μg 6 h before colonoscopy (total 580 μg). |
| Guo et al. | 4 L split-dose: 2 L on the evening before colonoscopy, then 2 L 4–6 h before colonoscopy. | Same 4 L PEG split-dose as control. | 290 μg before breakfast for 3 days before colonoscopy (total 870 μg). |
| Qi et al. | 3 L split-dose: 1 L at 20:00 on the day before colonoscopy, then 2 L 4–6 h before colonoscopy. | Same 3 L PEG split-dose as control. | 290 μg 30 min before breakfast on each of the 2 days before colonoscopy, plus 290 μg 8 h before colonoscopy (total 870 μg). |
| Zhang LJ et al. | 3 L split-dose: 1 L on the evening before colonoscopy, then 2 L 6 h before colonoscopy (completed within 2 h). | Same 3 L PEG split-dose as control. | 290 μg before breakfast for 3 days before colonoscopy (total 870 μg). |
| Ding et al. | 3 L split-dose: 2 L at 20:00 on the day before colonoscopy, then 1 L at 08:00 on the examination day. | Same 3 L PEG split-dose as control. | 290 μg on the day before colonoscopy and 290 μg on the examination day (total 580 μg). |
| Cheng X et al. | 3 L PEG regimen: 3 L compound PEG electrolyte powder (I) administered orally from 07:00 to 09:00 on the examination day. | 3 L split-dose PEG regimen: 1 L compound PEG electrolyte powder (I) from 18:00 to 20:00 on the day before colonoscopy, then 2 L from 07:00 to 09:00 on the examination day. | One capsule(290 μg) of linaclotide administered orally at 12:00 daily for 3 days before colonoscopy. (total 870 μg). |
| Zhang QL et al. | 2 L split-dose PEG regimen: 1 L compound PEG electrolyte powder III at 20:00 on the day before colonoscopy, then 1 L 4–6 h before colonoscopy; 200–250 mL every 10–20 min, completed within 1–2 h. | Same 2 L PEG split-dose regimen as control. | 290 μg linaclotide administered orally on an empty stomach at 05:00 on the examination day. |
| Sun et al. | 2 L PEG regimen: 2 L PEG solution administered 6 h before colonoscopy, 250 mL every 10–15 min within 2 h, followed by 160 mg simethicone. | Same 2 L PEG regimen as control, followed by 160 mg simethicone. | 290 μg linaclotide administered orally 8 h before colonoscopy. |
| Liu et al. | 3 L split-dose PEG regimen: 1 L PEG at 20:00 on the day before colonoscopy, then 2 L PEG 6 h before colonoscopy. | 2 L PEG administered 6 h before colonoscopy. | 290 μg linaclotide administered orally once daily for 2 consecutive days before colonoscopy. (total 580 μg). |

Supplementary Table S2. Regimens of PEG and linaclotide in the included randomized controlled trials. Doses, volumes, and administration schedules of PEG and linaclotide are summarized as reported in the original trials.


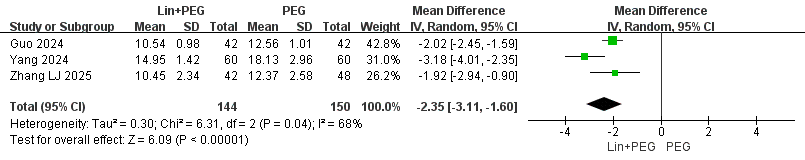



**Supplementary Figure 1: Forest plot comparing total procedure time between the Lin+PEG and PEG groups.**

**Supplementary Figure 2: Forest plot comparing the PDR between the Lin+PEG and PEG groups.**





**Supplementary Figure 3: Forest plot of subgroup analysis for total BBPS score based on patient characteristics.**

**Supplementary Figure 4: Forest plot of subgroup analysis for total BBPS score based on linaclotide dosage.**

**Supplementary Figure 5:** **Funnel plots for publication bias assessment. (A) Total BBPS score; (B) Left colon BBPS score; (C) Right colon BBPS score; (D) Transverse colon BBPS score; (E) Vomiting; (F) Abdominal pain; (G) Abdominal distension; (H) Nausea; (I) Total adverse events; (J) Procedure time; (K) PDR.**


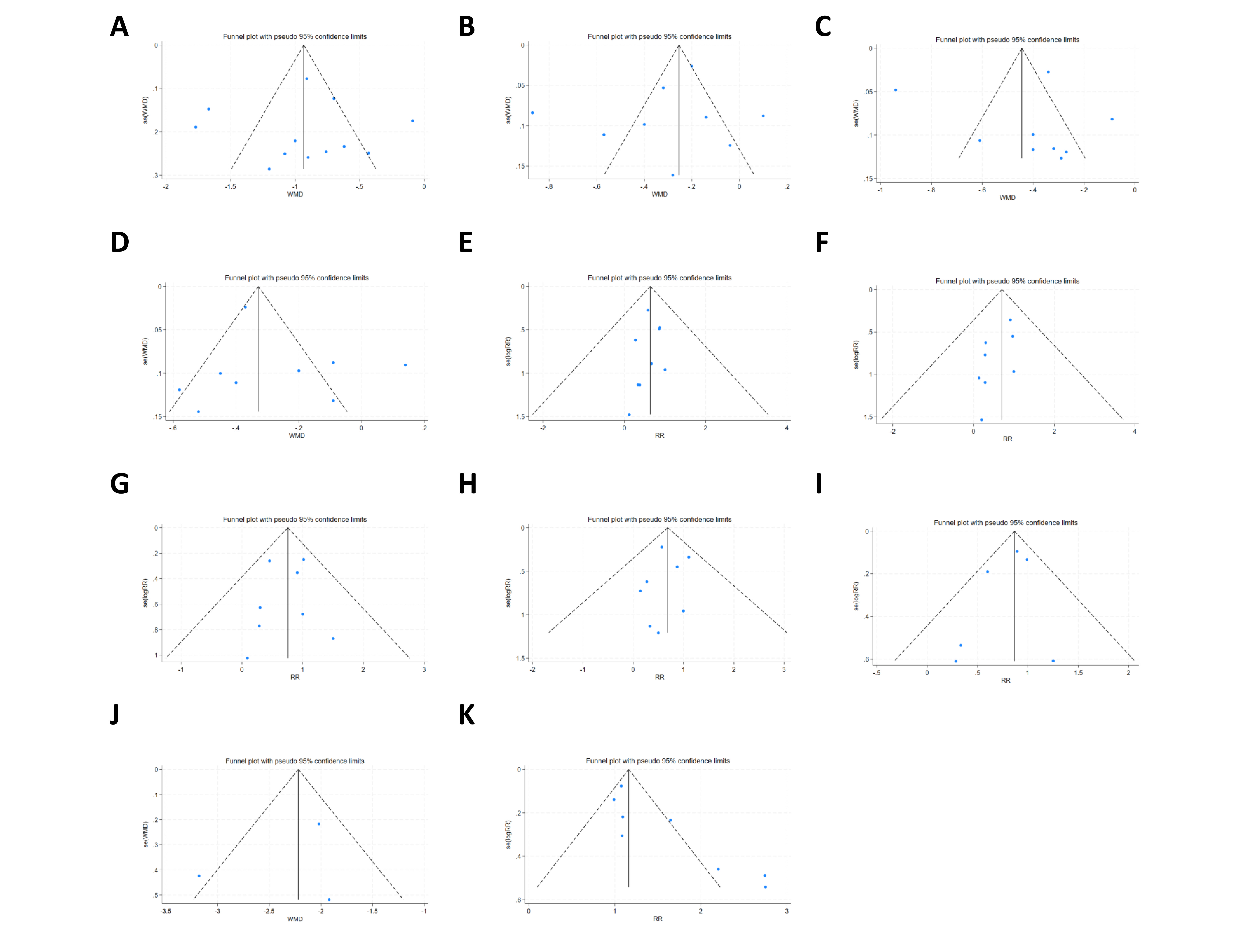

Supplement: Supplementary file 1 [file Supplementary_file_1.docx]
